# A data-driven approach to solve the RT scheduling problem

**DOI:** 10.1016/j.tipsro.2024.100282

**Published:** 2024-10-15

**Authors:** Mruga Gurjar, Jesper Lindberg, Thomas Björk-Eriksson, Caroline Olsson

**Affiliations:** a Medical Radiation Sciences, Institute of Clinical Sciences, Sahlgrenska Academy, University of Gothenburg, Sweden; b Department of Medical Physics and Biomedical Engineering, Sahlgrenska University Hospital, Gothenburg, Sweden; c Regional Cancer Centre West, Western Sweden Healthcare Region, Gothenburg, Sweden; d Department of Oncology, Institute of Clinical Sciences, Sahlgrenska Academy, University of Gothenburg, Sweden

**Keywords:** Radiotherapy Scheduling, Scheduling Imbalance, Diagnosis, Patient Delays, Automation

## Abstract

**Introduction:**

There is an increase in demand for Radiotherapy (RT) and it is a time critical treatment with a complex scheduling process. RT workflow is inter-dependent and involves various steps including pre-treatment and treatment-related tasks which adds to these challenges. Globally, scheduling delays are reported as one of the most common issues in RT. We aim to create and evaluate an automated strategy which generates a patient allocation list to assist the scheduling staff to create an efficient scheduling process.

**Methods and Materials:**

We used historical data from a large RT department in Sweden from January to December 2022 with 11–13 operational linear accelerators. The algorithm was developed in C# language. It utilizes patient and treatment-related characteristics including the patient timeline (referral date, preferred treatment start dates), booking category, diagnosis group and intent. Based on this, the algorithm assigns patient priority individually.

**Results:**

The algorithm’s output resulted in a scheduling list sorted by high to low patient priority per week. We evaluated the algorithm with historical manual allocations from the same year. The comparison between manual and algorithm allocations showed that the number of delayed patients reduced by 10 % in the algorithm suggestion with an average delay reduction of 2 weeks. Furthermore, the focus on patient-related characteristics resulted in diagnosis groups being better balanced.

**Conclusion:**

The algorithm’s ability to produce quick results may save significant time that the scheduling staff otherwise need to assess individual patient profiles. RT departments can incorporate such algorithms to accelerate their scheduling decisions and enhance their overall scheduling performance before going through major organizational changes.

## Highlights

• Algorithm-suggested allocations reduced the number of delayed patients by 10%
• The algorithm’s real-time adaptability reduced delays up to three weeks.
• The automated sorting strategy balanced allocations better between diagnosis groups.

## Introduction

Increasing number of cancer patients is resulting in a high demand for radiotherapy (RT). Considering the current level of limited RT resources, this demand can be difficult to meet, and patients may be facing long waiting times [1]. The RT process involves different tasks like molding, imaging, treatment planning, and quality assurance before the start of RT. Consequently, it is a complex task for the staff to schedule patients for treatment. To efficiently manage this, it is also important to consider external factors that affect the patient start date and other unpredictable aspects which can add to the complexity of the patient scheduling task [2]. However, published research on the RT scheduling problem and associated computational models has so far mainly focused on solutions based on resource capacity, resource availability, and staff allocations although it has been suggested that the patient throughput is more affected by inefficiency in patient scheduling than by inadequate resources [3].

To create a comprehensive solution for patient scheduling in RT, it is critical to acknowledge the patient distribution between different diagnosis groups and the treatment characteristics. Large diagnosis groups like breast cancer and prostate cancer generally require more attention since they are higher in number. It is a challenge for the scheduling staff to handle large volumes from these groups and inadvertently, the smaller or rare diagnosis groups can get overlooked unless they are scheduled for an emergency treatment. This imbalance in diagnosis groups may affect the scheduling task and increase the delays in the overall RT process [4], [5]. In addition, clinical factors such as patients going through co-ordinated treatments before RT may influence the start of RT. It has for instance been reported that the median interval time for breast cancer patients, between breast conserving surgery and RT, can be up to 32 weeks depending on the recovery of the patient [6]. Other breast patients may not require this long to start their treatment. It is important to note that timelines vary within and between every diagnosis group, as does routines for referral to RT, and these aspects will affect scheduling options differently.

RT is one of the most quantitative fields in healthcare, with most of the patient and appointment-related data processed and stored in the Oncology Information Systems (OIS) [7]. These data can be used outside the system as potential input to other applications or for historical analysis. It can also be used pre-emptively, to provide automated sorting strategies for effective scheduling of patients. Such strategies to use OIS data for this purpose have, however, not been much reported in the scientific literature.

The purpose of this work is to use a data-driven approach to design an algorithm which can simplify the scheduling task in RT and consequently assist in preventing the patient build-up in RT departments.

## Methods and Materials

### Data characteristics

The data used in the development of the algorithm were collected from a large RT department in Sweden through the OIS ARIA (Varian Medical Systems, Inc., Palo Alto, CA, U.S.A). The extracted data represent a 12-month period from January 2022 to December 2022. During this period, 11–13 linacs were operational and used for patient treatments. Note that linac services are evenly distributed throughout the year and that the operational schedule is adjusted to make time for this. The data included information on patient referrals (referral date, first preferred treatment start date, last preferred treatment start date, and actual treatment start date), diagnoses (ICD-10 codes), and treatment (intent, booking categories, and number of fractions). The work in this study was approved by the Regional Ethical Review Board in Gothenburg replaced with the Swedish Ethical Review Authority in 2019 (registration numbers: 841–16, T640-17, 2022–02683-02).

The extracted raw data included some inconsistencies (duplicated rows, blank rows, missing information) which were cleaned before being used for algorithm development and analysis. The identified diagnoses during the extracted period were sorted into to eight major diagnosis groups according to the categories of the Swedish national quality registry for RT data [8]. These are breast, central nervous system (CNS) and brain, gastro-intestinal, genitourinary, gynecology, head and neck, prostate, thorax. Detailed diagnosis distribution is given in the supplementary material.

**Fig. 1:**
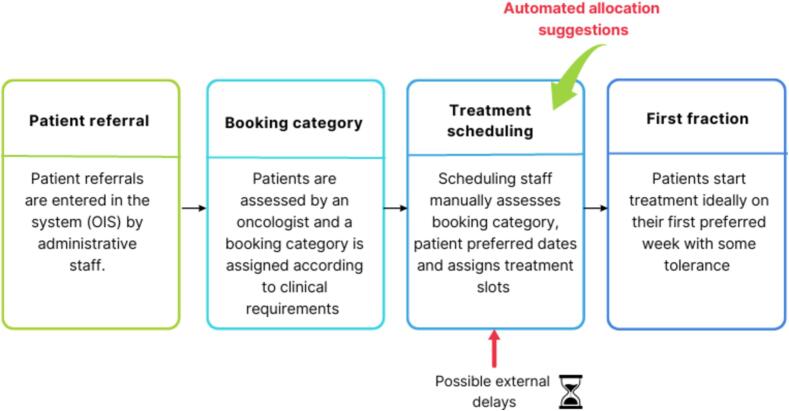
Data flow from referral to the scheduling of patients for radiotherapy. The algorithm provides an automated list of allocations based on the previous data flow. It also accounts for possible external delays including machine downtime, staff shortages which can affect the scheduling. Abbreviation: OIS- Oncology information systems.

Each patient referral is entered into the OIS by administrative staff. Further, oncologists assess individual patient profiles and assigns booking categories to the patient based on patient-treatment characteristics. The scheduling is generally reliant on the chosen booking categories and available resources. In Sweden, patients following Cancer Patient Pathways go through a pre-booked RT workflow and are automatically booked for RT ahead in time. The pathways are specific to each cancer diagnose and define the investigations and the acceptable time limits from when a suspicion of cancer is raised to the start of cancer treatment [9]. The cancer diagnoses receiving RT as a first treatment are high-malignant gliomas and GI-cancers as well as selected head and neck/esophagus/lung/gynecologic cancers receiving radio-chemotherapy. For this reason, the patients eligible for these pre-booked treatment slots are neither included in our analysis nor in the evaluation of the algorithm. An overview of the data flow from referral to the scheduling of patients for treatment is explained in Fig. 1.

### Algorithm development

**Fig. 2:**
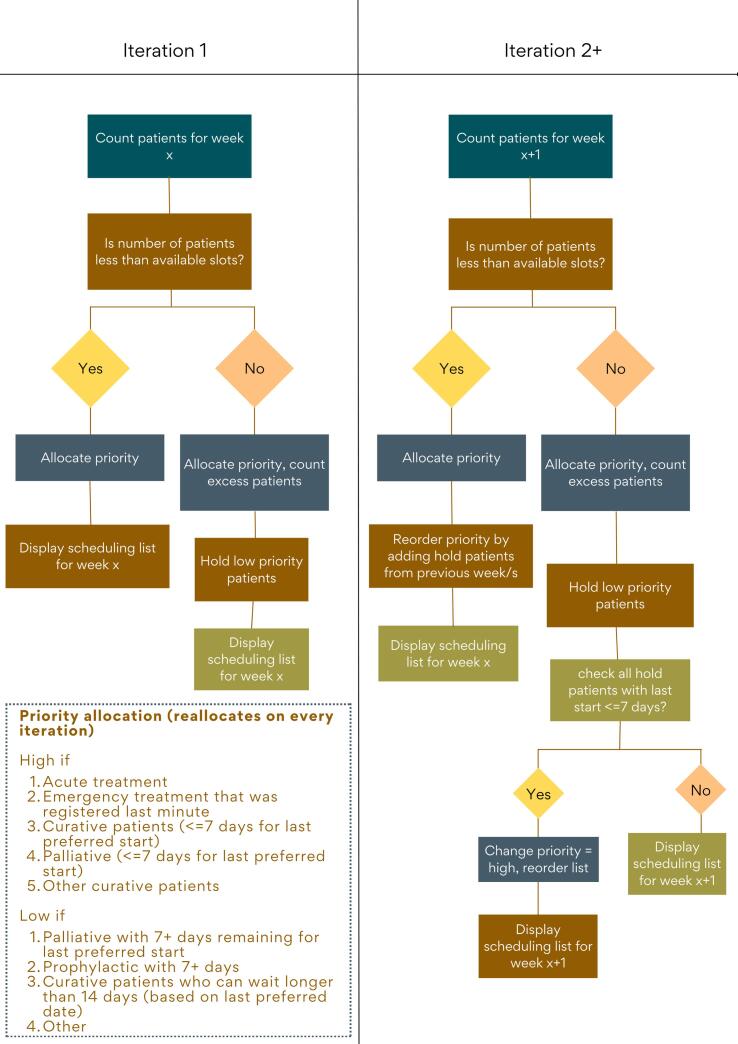
Process flow of the algorithm in simple terms. Note that the algorithm accesses an excel sheet with all patient and appointment data from 2022 as input. Weekly averages, formulae for assessing priority level one and two are performed by automated scripts within the excel sheet.

The algorithm was primarily built in C# (Visual Studio, Version 17.1, Microsoft, Washington, U.S.A) and included excel queries. It automates the principles of manual patient scheduling used by the scheduling staff in the RT department. The algorithm is based on the distribution of patients in different diagnosis groups and their preferred dates. It calculates and displays a list of ideal patient allocations per week based on historical data and available treatment slots in the following week. A treatment slot typically ranges between 30–45 min for first treatments and 15–20 min for the remaining treatments. The algorithm flow is presented in Fig. 2 and is explained in general terms below.

From the historical data, we identified the number of patients for each week having their preferred start date within those dates. This number reflects the number of appointments to be scheduled during the next week. Ideally, the algorithm provides an allocation suggestion according to the treatment slots available in the following week. Patients are initially sorted according to their clinical priority which is based on treatment intent and buffer time between their first and last preferred date. Emergency treatments, patients with curative intent and small buffer time are listed on top. These clinically high-priority patients are followed by clinically low-priority patients with palliative or prophylactic intent, or other patients with longer buffer time. Generally, curative patients with high priority (rapidly proliferating tumor types e.g. lymphomas) have a time target period to start treatment 2–4 weeks after referral, curative patients with lower priority (slowly proliferating tumor types e.g. breast- and prostate cancer) are to start treatment 6–8 weeks after referral, and palliative patients with non-acute/sub-acute/acute symptoms are to start treatment 1–3 weeks/3–7 days/1–2 days after referral.

**Fig. 3:**
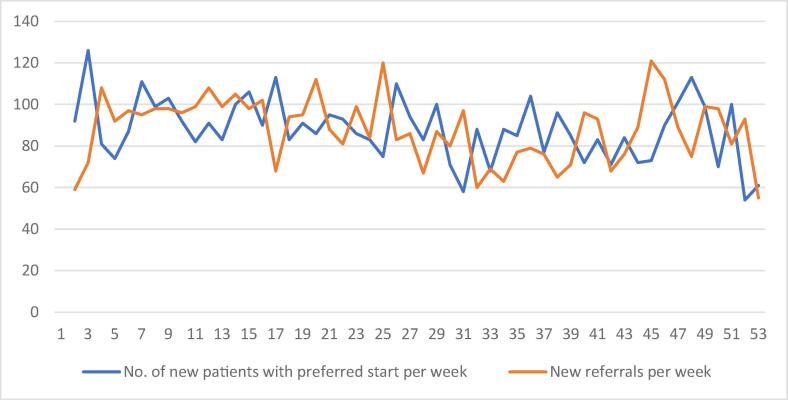
Fluctuations in patient referrals per week and number of patients with preferred start during that week, (according to their first preferred date) in 2022 at the radiotherapy department of Sahlgrenska University Hospital in Sweden. The data represent number of referrals that were registered at the department each week and total number of new appointments to be scheduled in that week according to the given target date (first preferred). Note that the new patient starts typically have a referral date in a previous week or months.

In real-time, the staff must also focus on new referrals registered at the department, particularly those who have shortly upcoming preferred start dates. Consequently, the number of patients who are scheduled to start can be lower or higher than available slots (Fig. 3). In situations where there are not enough slots available, patients on the lower end of the list with low priority from the algorithm’s perspective will be kept on hold and will be forwarded to the upper part of the list in the following weeks. The algorithm also checks if there are any delayed patients (on hold) from the previous weeks. If so, these patients are automatically sorted higher in the list. The process can be repeated at any desired time interval by scheduling the script on a computer through Microsoft Visual Studio. The output is presented as a sorted list (most urgent patients to be scheduled on top) and stored in an excel sheet. The current version includes patient information with unique identification number, diagnosis code, intent and other patient related details.

We evaluated the algorithm with weekly data during 2022 and comparisons were made monthly for the manual allocations and the suggested automated allocations by the algorithm. Descriptive statistics included patient allocations – high and low priority by algorithm’s assessment (mean values), delayed patients (mean values), and delay in weeks (mean values).

## Results

### Data characteristics

The raw dataset included a total of 5171 patients, out of which 41 entries (<1%) were inconsistencies. These included duplicated rows, blank rows and rows with missing patient information, which were removed. The cleaned dataset included a total of 5130 patients and, after excluding 559 patients from the pre-booked booking category (Cancer Patient Pathway), a total of 4571 patients were used for algorithm development. We identified 81 unique diagnoses during the extracted period, the largest being prostate with 1573 (35 %) patients, followed by breast with 1285 (29 %) patients in total. Afterwards, thorax group with 370 (8 %) patients and gastrointestinal group with 270 (6 %) patients. Number of patients in the remaining four groups (gynecology, CNS & brain, genitourinary and head and neck) ranged between 82 and 106 (3–5 %) patients per group. Due to the small numbers, these were combined to form an “other” diagnosis group. The number of patients treated with curative, palliative and prophylactic intent was 2467 (54 %), 2045 (45 %), and 50 (1 %), respectively. According to the historical data from 2022, the average number of available treatment slots for treatment start ranged between 90–98 per week acknowledging downtime/other factors.

**Fig. 4:**
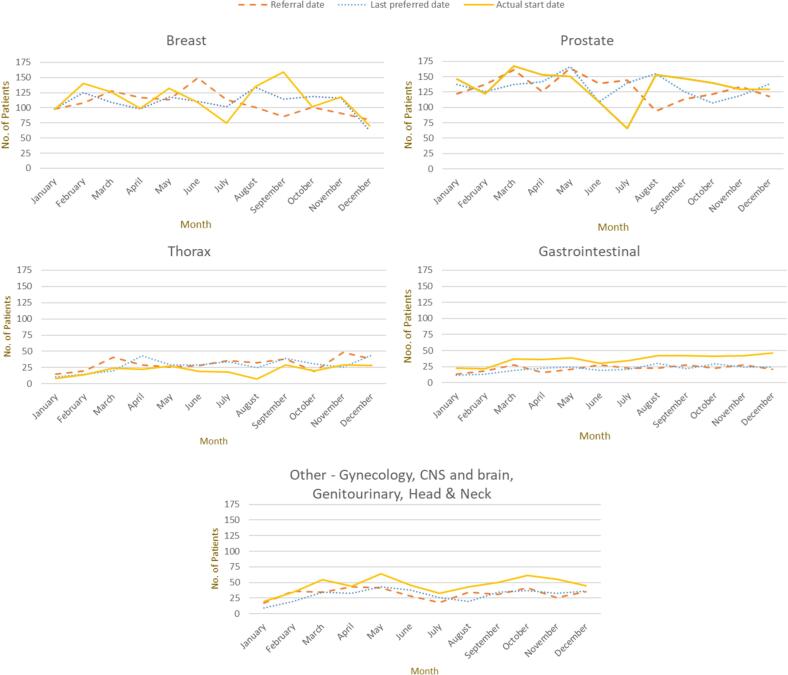
Referral dates, last preferred dates, and actual treatment start dates by diagnosis group for patients referred to treatment at the Sahlgrenska University Hospital in Sweden during 2022. Abbreviations: CNS- central nervous system. Note that some patients may have their referral date in previous months compared to their actual start date.

The monthly inflow of patients ranged between minimum of 331 (August) to maximum of 469 (March) patients during 2022. The largest two diagnosis groups every month were breast and prostate, accounting for on average 60 % of all patients. Fig. 4 shows the inflow of patients by referral, last preferred and actual start date for breast, prostate, thorax, gastrointestinal and other diagnosis groups. Note that the number of referrals per month does not directly reflect the number of patients who start treatment in the same month. The time between referral and last preferred date may be long for some patients due to coordinated treatments like surgery or chemotherapy.

### Algorithm output

**Fig. 5:**
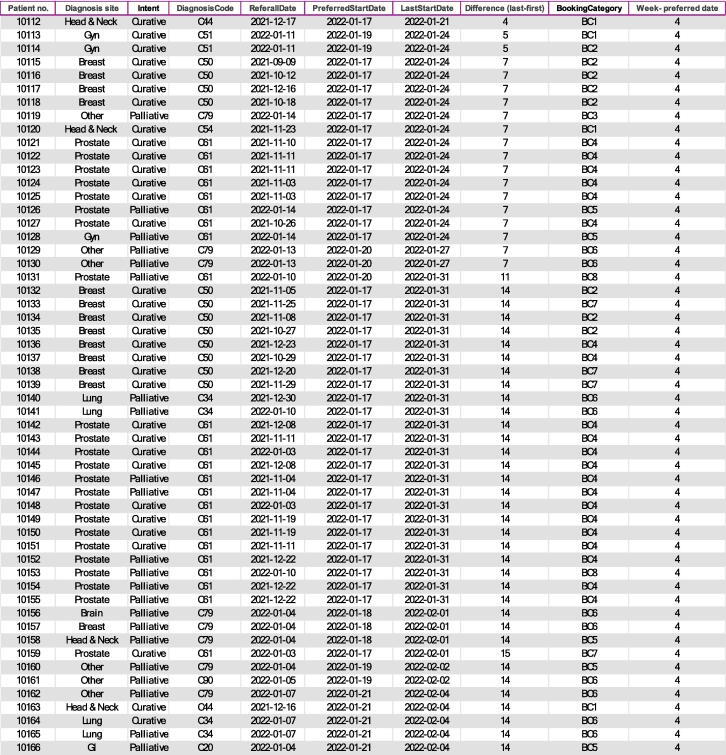
Screenshot from the output of the algorithm for patient scheduling (example Excel file version). The list of suggested patients is for week 4 in 2022 and patients are sorted by their last preferred start date. The suggested list also includes other treatment and patient related characteristics like diagnosis, intent, booking category. Note that the data have been somewhat manipulated to ensure no patient can be identified. Abbreviations: BC-Booking Category.

The output from the algorithm is given in Fig. 5. The output list is sorted according to patient priority based on treatment intent and difference between first and last preferred date. Based on these characteristics, scheduling staff can make an informed decision when scheduling patients for RT.

### Comparisons between manual and automated allocation of patients

Table 1 shows the comparisons between number of patients who were manually allocated by the scheduling staff and patient allocation suggestions by the algorithm in the year 2022. To ensure consistency and fair assessment between the performance of the automated strategy and the manual process, the output from the algorithm was adjusted to match the same number of patients as the manually allocated number each month.

**Table 1:** Comparison table for manual scheduling and algorithm suggestion for the year 2022. Parameters used for the comparison are based on number of patients scheduled and averaged delays.

2022	Jan		Feb		March		April		May		June		July		Aug		Sept		Oct		Nov		Dec		TOTAL	
**Metric**	M	A	M	A	M	A	M	A	M	A	M	A	M	A	M	A	M	A	M	A	M	A	M	A	M	A
**Number of Allocations**	**379**	**379**	**392**	**392**	**452**	**452**	**388**	**388**	**438**	**438**	**333**	**333**	**240**	**240**	**405**	**405**	**449**	**449**	**374**	**374**	**395**	**395**	**326**	**326**	4571	4571
− High Priority	203	215	222	241	240	266	195	213	253	271	185	191	154	164	264	281	300	314	216	233	231	239	179	189	2642	2817
− Low Priority	176	164	170	151	212	186	193	175	185	167	148	142	86	76	141	124	149	135	158	141	164	156	147	137	1929	1754
**Delayed Patients**	**54**	**50**	**49**	**44**	**51**	**49**	**44**	**49**	**56**	**51**	**66**	**57**	**81**	**67**	**65**	**66**	**45**	**41**	**39**	**33**	**41**	**33**	**45**	**39**	636	579
− High Priority	33	11	29	13	26	18	21	22	29	25	38	15	48	19	30	17	21	21	19	16	23	17	21	19	338	213
− Low Priority	21	39	20	31	25	31	23	27	27	26	28	42	33	48	35	49	24	20	20	17	18	16	24	20	298	366
Delay (Weeks)	2 ± 1	1 ± 1	3 ± 1	2 ± 1	2 ± 1	2 ± 1	2 ± 2	2 ± 1	3 ± 2	2 ± 2	4 ± 1	2 ± 2	6 ± 2	4 ± 1	6 ± 3	5 ± 2	5 ± 1	4 ± 1	3 ± 2	3 ± 1	3 ± 1	2 ± 1	2 ± 2	2 ± 1	3 ± 1	2 ± 2

Abbreviations: M- Manual allocation, A- Algorithm output,

Note that “High” and “Low” priorites in this context are based on the algorithm's assessment and do not necessarily reflect clinical priority levels.

Overall, the algorithm suggested 7 % more high priority patients than manually scheduled. The number of delayed patients was reduced by 10 % in the algorithm suggestion with an average delay reduction of 2 to 3 weeks. Monthly comparisons revealed a consistent pattern with more high priority patients being suggested by the algorithm while for manual allocation, high and low priority patients in most cases, were equally distributed for the allocation part. The number of delayed patients was reduced by 4 to 14 patients per month (January and July, respectively). Overall, there was no noticeable difference observed for March, April, October, and December. However, the delay for the rest of the months was reduced by an average of 1 to 2 weeks.

Comparisons between manual allocations and algorithm suggestions for specific diagnosis groups are shown in Table 2. Manual allocation resulted in a greater number of patients scheduled for larger diagnosis groups (breast and prostate) while the algorithm suggested lower numbers for these groups. In contrast, the algorithm suggested greater number of patients compared to manual for smaller diagnosis groups namely, GI, thorax and other with a difference of 15 %, 41 % and 8 % respectively. The number of delayed patients for smaller diagnosis groups was reduced by 24 % for both GI and thorax and 16 % for other groups.

**Table 2:** Comparison table for manual scheduling vs algorithm suggestion in terms of different diagnosis groups for 2022.

2022	Q1		Q2		Q3		Q4		Total	
	Manual	Auto	Manual	Auto	Manual	Auto	Manual	Auto	Manual	Auto
**Breast**	363	332	340	327	369	350	290	297	1362	1306
Delayed Patients	32	39	37	42	35	41	29	27	133	149
**Prostate**	435	400	412	417	366	322	398	365	1611	1504
Delayed Patients	29	35	30	24	37	35	19	23	115	117
**GI**	39	44	60	66	66	73	62	77	227	260
Delayed Patients	15	12	15	11	15	14	19	13	64	50
**Thorax**	46	46	68	101	54	98	77	101	245	346
Delayed Patients	15	11	23	17	29	24	24	19	91	71
**Other**	57	63	100	114	77	80	102	106	336	363
Delayed Patients	21	16	16	14	19	18	18	15	74	63

Abbreviations: M- Manual allocation, A- Algorithm output suggestion, Q-quarter.

### Sensitivity analysis

**Fig. 6:**
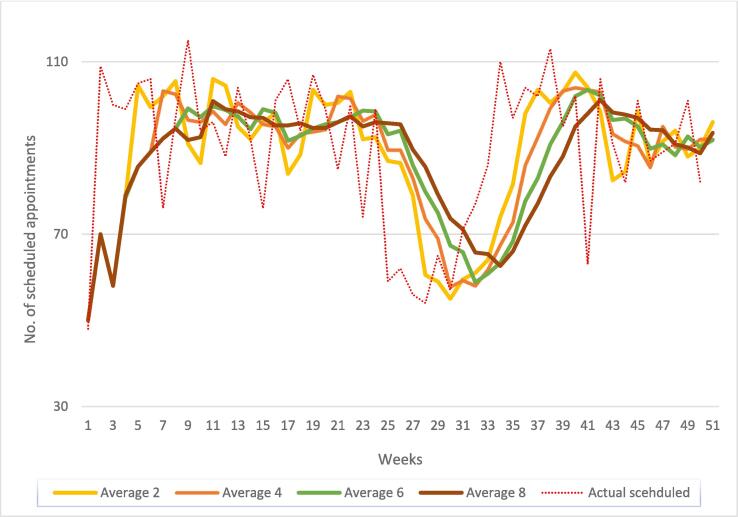
Sensitivity analysis of historical averages of scheduled patients by weeks. The graph shows trend lines for the number of scheduled patients given 2-, 4-, 6- and 8-week historical averages in 2022. The red dotted line indicates the actual number of scheduled appointments that week. Note that the peaks typically relate to Swedish holidays such as school winter break and Easter holidays (week 7, 15), summer vacations (weeks 23–31), and Christmas holidays (week 1, 51). (For interpretation of the references to colour in this figure legend, the reader is referred to the web version of this article.)

We performed a sensitivity analysis to understand the impact of variability in average historical appointments on the scheduling process. In our experience, historical average reflects potential operational incidents such as staff or resource capacity fluctuations or external events like organizational changes. We looked at historical average of 2, 4, 6 and 8 weeks. The results given in Fig. 6 showed a consistent pattern through week 5 to week 26, suggesting a stable schedule and regular operational period with few fluctuations in staff or resources. However, number of average appointments rapidly dropped between week 26 and week 34. This deviation was caused by lower staff capacity during the summer vacation period.

There was a notable difference between 2-week and 8-week historical averages. The 8-week average underestimated the actual number of scheduled appointments while the 2-week average closely depicted the periodic variation. The 6-week average was sensitive to long term trends and failed to capture recent fluctuations. In our opinion, the 4-week average maintained balance between short- and long-term trends and minimized the impact of outliers.

## Discussion

Using real-world scheduling data from the OIS, we developed and evaluated a sorting algorithm to assist in the scheduling of patients for treatment. Our strategy automates the principles of the manual scheduling process and balances individual patient-treatment characteristics with available treatment slots. In comparison with manual allocations, the algorithm resulted in less overall waiting time by changing the distribution between low and high priority patients. Unlike manual scheduling, the algorithm prioritizes patients based on preferred start dates and not solely on clinical priority, allowing more flexibility to schedule patients with tighter deadlines and making the process easier with potentially more available slots to choose from. More high-priority patients (all patients with tighter deadlines) were scheduled, which is in fact a strategic allocation since patients with longer deadlines can afford to wait longer. For certain months, the number of allocations remained comparable, however, during crucial periods with reduced capacity (e.g. vacation), the algorithm-suggested allocations were according to the historical averages and seasonal changes resulting in less patients being delayed. Quarterly comparisons between diagnosis groups revealed that some patients from the larger diagnosis groups could wait longer while patients from other smaller diagnosis groups could be scheduled earlier. In addition, to improve patient allocations and reduce the number of delayed patients, the algorithm also produced quick results and has the potential to make the scheduling task more time efficient for the scheduling staff.

RT is known to be one of the most time-critical disciplines and central to the RT workflow is prompt scheduling of patients [4]. Patient delays and long wait times, however, is noted as one of the most common issues in RT [10], [11]. A frequently used approach to manage patient delays includes strategies based on resource management. Other existing strategies have focused on predicting patient delays or identifying bottlenecks while computational approaches to simplify scheduling have rarely been investigated. A study from 2017 investigated a discrete event simulation model on the entire workflow to target reduction in delays [12]. Resource capacity levels were fluctuated to identify bottlenecks in the process and the outcome was measured in ready-to-treat treatment time (RTTT). The authors found that employing one additional full-time dosimetrist would reduce the delay and keep RTTT under 14 days after pre-treatment. This study demonstrates how to reach optimal resource performance levels, however, implementing administrative changes may take a long time. Incorporating data-driven solutions similar to our proposed strategy can be faster as such tools do not require extensive approvals. Another study in 2017 used machine learning to predict individual patient’s wait time and provide accurate estimates through a mobile application for patients and staff [13]. Main features used in this model were number of fractionations, treatment difficulty, time for pre-treatment tasks. However, the authors could not determine if estimating delays would in fact reduce the delays overall. Another limitation discussed in both studies was the inability to capture real-time changes. Our work addresses this limitation by allowing the algorithm to capture such modifications directly from the database. For example, patients who cannot initiate treatment due to some circumstances are removed from the system and will disappear from the scheduling list. Employing both real time updates and historical analysis results in more a scenario-based allocation suggestion.

Patient scheduling problems have also been linked to imbalances between diagnosis groups [14]. However, this scheduling issue in our experience is generally overlooked by RT departments and existing research in this area is only limited to resolving diagnosis specific scheduling issues. A PubMed search on June 15th, 2024, on different combinations of “scheduling”, “imbalance”, “diagnosis”, “delays” and “radiotherapy” gave only one relevant hit. This recent qualitative study in Norway reported that the scheduling staff is torn between meeting scheduling targets and managing resources and capacities [15]. The staff, although aware of the delays and imbalances between diagnoses, must find new strategies in real-time to solve various scheduling issues daily. Furthermore, manual assessments of patients, resources, and urgency adds additional stress in maintaining equal ratios between rare and common diagnoses. This study also expresses concern about the importance of understanding the requirements of frontline workers in efforts to realise required organisational changes [15]. In our work, focus on patients and treatment related characteristics resulted in diagnosis groups being better balanced without any additional measures. Scheduling suggestions for the two largest diagnosis groups breast and prostate cancer lowered while numbers for smaller diagnoses increased. This maybe due to staff shortages and patient backlogs experienced when scheduling patients manually. One must also keep in mind that our presented results are based on the historical data from 2022 and that scheduling suggestions may differ depending on incoming referrals in the future.

For seamless adaptation of new strategies, it is important to consider necessary changes that are required in work practices. The algorithm is developed under the assumption that the scheduling staff works in a collaborative approach. This can be one of the limitations since number of scheduling staff and their responsibilities differ in every RT department. In some cases, the scheduling staff may work independently on specific diagnosis groups. This can lead to problems in accurately tracking the allocations from the suggested list. To resolve this, the algorithm needs to be tweaked to support individual staff requirements. A study published in 2023 explains the importance of including ***margin for changes*** when designing systems related to appointment scheduling [16]. Due to unexpected situations like emergency appointments, sudden machine downtime, the scheduling staff may need to take strategic scheduling decisions which are not in line with the suggested output. In this case, If the algorithm is not run regularly, it may affect the performance of future runs. This can be solved during practical implementation by allowing the user to edit the patient list through a user interface. This will potentially increase the scenario-based adaptability of the algorithm. The algorithm relies on the information that is available in the OIS and might not result in an accurate output if there is a situation where significant amount of information is missing. To mitigate the occurrence of such circumstances, the staff may be encouraged to promptly enter patient data and conduct routine surveys to track any issues or disturbances in the system. In the next version, we aim to add a data overview functionality which will record the percentage of missing information and report it when required. Finally, future developments of the algorithm could include functionality for scheduling additional parts of the RT workflow besides the treatment start. Given that information about resources for each task can be quantified (e.g., availability of imaging device time slots for the pre-treatment stage), users of the tool could have the option to select which part of the RT workflow to schedule with the algorithm output providing task-specific scheduling suggestions.

In conclusion, the scheduling of patients in RT is a multifaceted process that is impacted by various external aspects. The current solution provides a way to make scheduling of patients easier and reduce the number of delayed patients at modern large RT departments. The presented algorithm also maintained overall balance between diagnosis groups. While the proposed strategy suggests significant scheduling improvements in theory, a practical implementation may require adjusting the current version to fit in different work practices. By harnessing the power of historical data and simple computational strategies, RT departments can incorporate such tools and enhance their workflow performance before having to go through major organizational changes.

## Declaration of competing interest

The authors declare that they have no known competing financial interests or personal relationships that could have appeared to influence the work reported in this paper.
