# Supplementary material for: A data-driven approach to solve the RT scheduling problem
Source: Tech Innov Patient Support Radiat Oncol. 2024 Oct 15;32:100282. doi: 10.1016/j.tipsro.2024.100282 (PMC11533699; doi:10.1016/j.tipsro.2024.100282)
Supplement: Supplementary Data 1 [file mmc1.docx]

# Supplementary material

Table 1- The diagnosis distribution according to Swedish National Quality Registry is given for each diagnosis group with their corresponding diagnoses represented by ICD-10 codes.

| Prostate group (1) | Breast group (2) | Thorax (4) | Central Nervous System and brain (6) | Genito-  Urinary (8) | Gynecology (8) | Head and Neck  (10) | Gastro-  Intestinal  (15) | Miscellaneous  (46) |
| --- | --- | --- | --- | --- | --- | --- | --- | --- |
| C61 | C50 | C34 | C70 | C60 | C51 | C01 | C15 | C40 |
|  | D05 | C37 | C71 | C62 | C52 | C02 | C16 | C41 |
|  |  | C38 | C72 | C64 | C53 | C03 | C17 | C44 to C47 |
|  |  | C39 | D32 | C65 | C54 | C05 | C18 | C49 |
|  |  |  | D33 | C66 | C55 | C06 | C19 | C73 to C86 |
|  |  |  | D43 | C67 | C56 | C07 | C20 | C88 |
|  |  |  |  | C68 | C57 | C09 | C21 | C91 to C94 |
|  |  |  |  | D41 | D06 | C30 | C22 | C96 |
|  |  |  |  |  |  | C32 | C23 | D02 |
|  |  |  |  |  |  | C69 | C24 | D03 |
|  |  |  |  |  |  |  | C25 | D07 |
|  |  |  |  |  |  |  | C26 | D11 |
|  |  |  |  |  |  |  | C48 | D15 |
|  |  |  |  |  |  |  | D01 | D16 |
|  |  |  |  |  |  |  | D37 | D21 |
|  |  |  |  |  |  |  |  | D24 |
|  |  |  |  |  |  |  |  | D35 to D38 |
|  |  |  |  |  |  |  |  | D44 |
|  |  |  |  |  |  |  |  | D47 |
|  |  |  |  |  |  |  |  | D48 |
|  |  |  |  |  |  |  |  | E05 |
|  |  |  |  |  |  |  |  | H35 |
|  |  |  |  |  |  |  |  | M61 |
|  |  |  |  |  |  |  |  | Q28 |
|  |  |  |  |  |  |  |  | Q85 |

Following are the names of the nine most common booking categories.

1. Cancer Patient Pathways
2. Coordinated treatments
3. Gold markers
4. Mammillary irradiation
5. Palliation
6. Post-operation
7. Single treatment
8. Special treatment
9. Standard appointments

Note that the booking categories from 1 to 9 are listed alphabetically, and do not represent a ranking from 1-9 (high to low priority).
